# The major CD8 T cell effector memory subset in the normal and *Chlamydia trachomatis*-infected human endocervix is low in perforin

**DOI:** 10.1186/1471-2172-13-66

**Published:** 2012-12-07

**Authors:** Joyce A Ibana, Leann Myers, Constance Porretta, Maria Lewis, Stephanie N Taylor, David H Martin, Alison J Quayle

**Affiliations:** 1 Microbiology, Immunology and Parasitology Department, Louisiana State University Health Sciences Center, New Orleans, Louisiana, 70112, USA; 2 Section of Pulmonary/Critical Care, Department of Medicine, Louisiana State University Health Sciences Center, New Orleans, Louisiana, 70112, USA; 3 School of Public Health and Tropical Medicine, Tulane University, New Orleans, Louisiana, 70112, USA; 4 Section of Infectious Diseases, Department of Medicine, Louisiana State University Health Sciences Center, New Orleans, Louisiana, 70112, USA

## Abstract

**Background:**

The local tissue microenvironment plays an important role in the induction, homing, maintenance and development of effector functions of T cells. Thus, site-specific differences in phenotypes of mucosal and systemic T cell populations have been observed. *Chlamydia trachomatis* most commonly infects the endocervix in women, yet little is known about *Chlamydia*-specific effector T cell immunity at this unique mucosal site. Our previous flow-cytometry-based study of cervical-cytobrush retrieved cells indicated that CD8 T cells are significantly increased in the *C. trachomatis*-infected human endocervix. The cytolytic function of CD8 T cells is important in the protective immunity against many intracellular pathogens, and requires the cytolytic granule perforin to facilitate the entry of other molecules that mediate the lysis of target cells. Determination of perforin expression of the CD8 T cell population in the endocervix would therefore provide insights on the granule-mediated cytolytic potential of these cells at this site.

**Results:**

Our histological data revealed that *C. trachomatis*-infected tissues have significantly higher numbers of CD3 and CD8 T cells compared to non-infected tissues (p<0.01), and that the majority of CD8^+^ cells do not express perforin *in situ*. A subsequent flow cytometric analysis of paired blood and endocervix-derived cells (n=16) revealed that while all the CD8 T cell subsets: naïve, effector memory (T_EM_), central memory (T_CM_) and terminally differentiated effector memory (T_EMRA_) can be found in the blood, the endocervix is populated mainly by the T_EM_ CD8 T cell subset. Our data also showed that perforin expression in the T_EM_ population is significantly lower in the endocervix than in the blood of *C. trachomatis* positive women (n=15; p<0.0001), as well as in *C. trachomatis*-negative individuals (n=6; p<0.05). Interestingly, our *in vitro* co-culture study suggests that the exposure of HeLa 229 cervical epithelial cells to IFN gamma could potentially induce a decrease in perforin content in CD8 T_EM_ cells in the same microenvironment.

**Conclusions:**

The low perforin content of CD8 T_EM_ cells in the endocervix, the local site of *C. trachomatis* infection in women, may reflect the unique immunological environment that balances immune protection against sexually transmitted infections and immune- tolerance to support conception.

## Background

CD8 T cells are key cellular components in the control of many intracellular microbial infections via their cytolytic function. *Chlamydia trachomatis* serovars D-K are intracellular bacteria that infect the columnar epithelial cells of the genital tract. Epithelial cells can present antigens in the context of MHC class I and activate a CD8 T cell immune response. Thus, investigation of the CD8 T cell cytolytic response to *C. trachomatis* infection is important as it could reveal a mechanism by which the bacterium is deprived of its intracellular niche. The major CD8 T cell cytolytic pathway involves the perforin and granzyme mediated induction of apoptosis [1,2]. Perforin mediates the delivery of granzymes to target cells by homopolymerization in the plasma membrane in a Ca^2+^ dependent manner producing pores that acts as a channel for granzyme entry [3,4]. Perforin is suggested to be necessary in CD8 T cell cytolytic activity, as perforin deficient mice have reduced efficiency in controlling viral infection [5].

During the course of infection, CD8 T cells differentiate and this is accompanied by changes in the expression of surface markers and functional capacity [6]. Naïve T cells are activated when they encounter their specific peptide-MHC complexes on professional antigen presenting cells [7]. A memory T cell differentiation pathway has been established by the group of Sallusto and Lanzavecchia, and others, whereby subsequent to antigen encounter, T cells proliferate and undergo phenotypic changes that modify their tissue homing properties [8-10]. Antigen-specific cells possessing a naïve-like phenotype (CD45RA^+^ CCR7^+^) are recruited into a pre-memory subset before reaching the central memory (T_CM_) and effector memory (T_EM_) stages that are characterized as CD45RA^-^CCR7^+^ and CD45RA^-^CCR7^-^ respectively. Eventually, these memory T cells reach a terminally differentiated effector stage (T_EMRA_) characterized as CD45RA^+^ CCR7^-^. Progression of a T cell through these subsets is associated with the acquisition of effector function and loss of proliferative potential *[ibid]*. Effector CD8 T cells acquire the capacity to migrate to extra lymphoid sites to sites of infection, and deliver perforin and granzyme at the immunological synapse to kill infected target cells [11]. CD8 T cell differentiation in response to infection is thus characterized by the acquisition of immunological properties that allow them to successfully clear intracellular pathogens.

Evidence that specific tissue microenvironments significantly influence CD8 T cell phenotype and function is accumulating. For example, Masopust *et al.*, illustrated that the gastrointestinal microenvironment promotes differentiation of a unique memory CD8 T cell population, and that CD8 T cells can switch phenotypes with changes in anatomic location [12]. The influence of the anatomic micromilieu was also illustrated by a study by Shacklett *et al.*, who demonstrated that unlike those in peripheral blood, CD8 T cells resident in the gastrointestinal tract of normal macaques were low in perforin. The absence of perforin in normal GI tissue was interpreted to be a mechanism by which the anatomical integrity of this mucosal site is protected, though it provides advantages for pathogens [13].

The female genital tract (FGT), like the GI tract, is also frequently exposed to foreign antigens, including commensal and pathogenic microorganisms. Furthermore, as a reproductive site, the FGT must tolerate foreign antigens to support conception. We thus hypothesized that CD8 T cells in the endocervix may also have limited cytolytic potential compared to their peripheral counterparts. Therefore, to investigate the phenotype of CD8 T cells in the FGT in the absence and presence of *C. trachomatis* infection, we sampled endocervical CD8 T cells from women to characterize the immune cell population, and assess the cytolytic potential of CD8 T cells at this site. In addition, using an *in vitro* approach, we further tested whether the presence of IFN gamma in a microenvironment could influence the perforin expression of CD8 T_EM_ cells.

## Results

### CD8 T cells infiltrate the human endocervix during *C. trachomatis* infection

**Figure 1:**
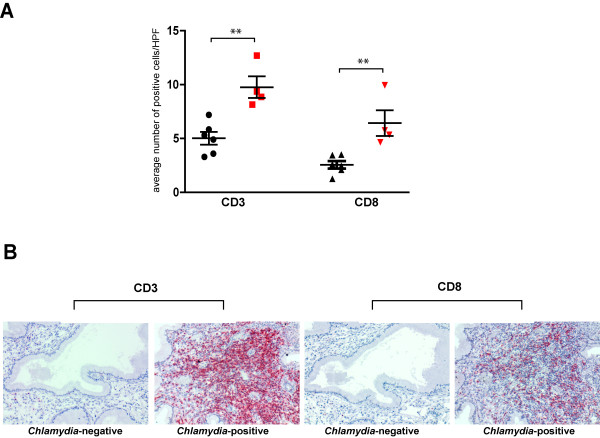
**T cell infiltrates in endocervical tissue sections.** (**A**) The number of CD3^+^, CD8^+^ cells in six *C. trachomatis*-negative (black) and four *C. trachomatis*-positive (red) endocervical tissues were assessed by immunohistochemistry. T cell counts represent the mean number of positive cells per high power field (HPF). Mean number of positive cells were derived from examination of twenty high power fields for each tissue sample. Statistical analysis was performed using Student’s t-test and confirmed by Wilcoxon Rank Sum test. Asterisks denotes p<0.01. (**B**) Representative *C. trachomatis*-negative and *C. trachomatis*-positive endocervical tissues stained with anti-CD3 and anti-CD8 antibodies. Red stain indicates CD3 or CD8 positive cells.

**Figure 2:**
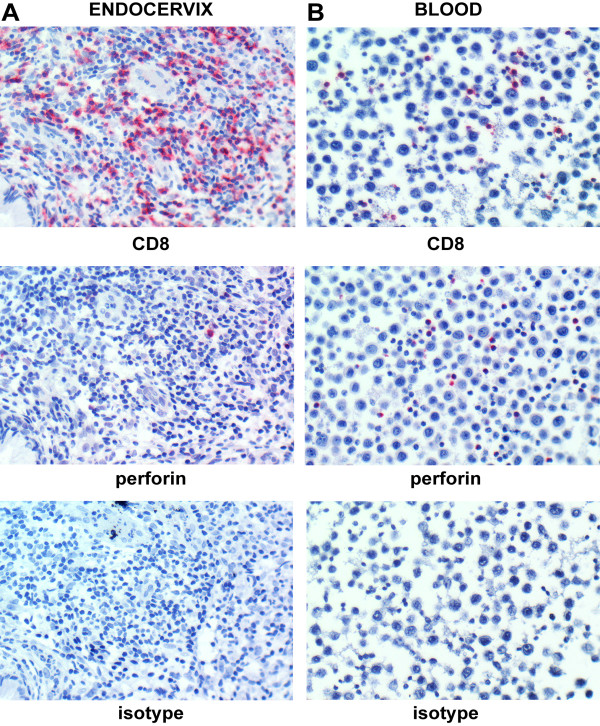
**Immunohistochemical staining profile of a representative *C. trachomatis*-positive tissue sample.** (**A**) CD8, perforin and isotype-control staining pattern of serial sections of a representative paraffin-embedded endocervical tissue from the *C. trachomatis*-infected population (n=4), (**B**) Sections of a cell pellet consisting of peripheral blood mononuclear cells (PBMCs) cushioned with HeLa 229 cervical epithelial cells is shown as positive staining control. Sections stained with isotype control antibodies were used as negative control. Vulcan fast red stain indicates CD8 or perforin positive cells. All tissue and cell pellet sections were counterstained with hematoxylin. The majority of CD8 T cells that infiltrate the *C. trachomatis*-infected tissues do not express detectable levels of perforin.

Consistent with our previous findings with cytobrush-retrieved endocervical T cells, immunohistological staining for CD3 and CD8 T cell infiltrates in six *C. trachomatis-*negative and four *C. trachomatis*-positive banked endocervical tissues demonstrated that the number of CD3^+^ and CD8^+^ T cells is significantly higher in *C. trachomatis*-positive than in *C. trachomatis*-negative endocervical tissues (p <0.01 for both comparisons) (Figure 1). Interestingly, when we stained serial sections of endocervical tissues for CD8 and perforin, we observed that the majority of CD8 positive cells were perforin negative (Figure 2). Quantitative analysis of perforin-expressing CD8 T cells, however, was limited by the immunohistological single staining technique’s inability to differentiate these cells from other immune cells that also express perforin, such as eosinophils, basophils, mast cells and NK cells. Thus, while our immunohistochemical data provided significant insights on the make-up of T cell infiltrates and suggested that CD8 T cells expressed limited amounts of perforin, we recognized that a multiparameter flow cytometric analysis would be needed to support and extend these observations.

### The CD8 T cell repertoire in the human endocervix is distinct from the periphery

**Figure 3:**
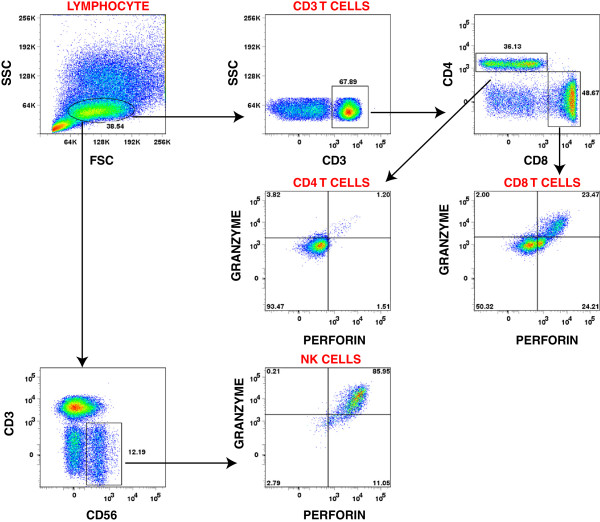
**The gating strategy used to identify the different immune cell populations.** Isolated cells from blood and endocervix were first gated based on forward scatter and side scatter. T cells were then identified based on CD3 positivity and NK cells were identified based on CD3^-^CD56^+^ profile. From the T cell CD3^+^ gate, CD4^+^ and CD8^+^ T cells were delineated. Following immune cell identification, perforin and granzyme expression were assessed.

**Figure 4:**
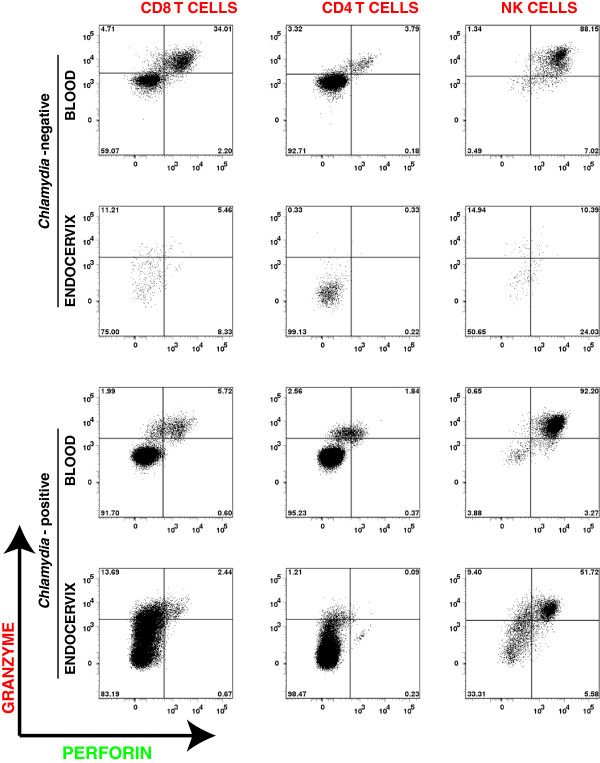
**Relative perforin and granzyme staining of CD8, CD4 T cells and NK cells.** Representative dot-plots of perforin and granzyme expression are shown for each immune cell types derived from the blood and endocervix of representative *C. trachomatis*-negative and *C. trachomatis*-positive women. The relative expression pattern across blood and endocervix from the same patient demonstrate that the perforin staining is lowest in CD4 T cells and highest in NK cells (CD4 T cells < CD8 T cells < NK cells).

To further analyze the endocervical immune cell repertoire, we performed multiparameter flow cytometric analyses to determine the mononuclear leukocyte types in isolated peripheral blood mononuclear cells (PBMC) and cytobrush-retrieved endocervical cells from 15 *C. trachomatis-*infected and 6 uninfected young women attending the Delgado Clinic. The gating strategy utilized in this study is shown in a representative analysis flow chart shown in Figure 3. Utilizing this strategy, we identified the lymphocyte population based on forward (FSC) and side-scatter (SSC) profile, from which the NK cell, CD4 T cell, and CD8 T cell subpopulations were delineated. We then assessed the perforin and granzyme expression in each of these cell types from paired samples obtained from the blood and endocervix. NK cells and CD4 T cells were utilized as cellular controls, while granzyme B was utilized as an intracellular staining control. Perforin and granzyme B expression of the different immune cells from the blood and endocervix of a representative *C. trachomatis*-negative and *C. trachomatis*-positive women is shown in Figure 4. Consistent with previous reports, we observed that perforin expression is relatively low in CD4 T cells, and high in NK cells [14,15].

**Figure 5:**
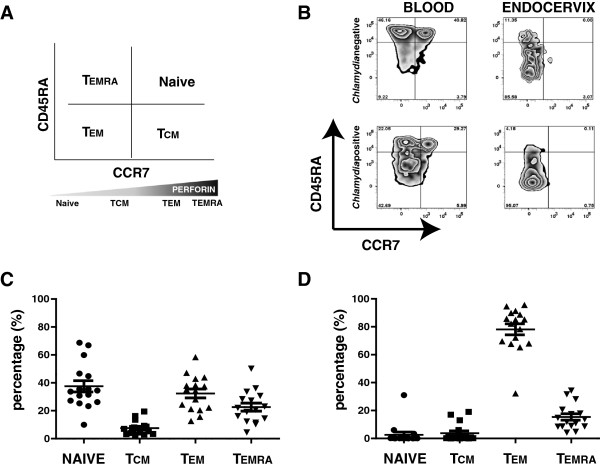
**CD8 T cell subpopulation distribution in the blood and endocervix.** (**A**) A diagram of Sallusto and Lanzavecchia’s classification scheme of CD8 T cells based on CD45RA and CCR7 expression and relative perforin expression of the different subpopulation, (**B**) distribution of the different CD8 T cell subpopulations in a representative *C. trachomatis*-negative and *C. trachomatis*-positive women (C-D) Distribution of different CD8 T cells in the blood (**C**), and in the endocervix (**D**) of 16 women (n=16). Using flow cytometric analyses, CD8 T cells were identified in a population of cells from PBMC and endocervical cells by gating for CD3^+^CD8^+^ cells. CD8 T cells were further characterized based on CD45RA and CCR7 expression. Naïve cells are CD45RA^+^ CCR7^+^, central memory (TCM) cells are CD45RA^-^CCR7^+^, effector memory (T_EM_) cells are CD45RA^-^ CCR7^-^, and terminally differentiated effector memory (T_EMRA_) cells are CD45RA^+^ CCR7^-^. The majority of CD8 T cells in the endocervix are T_EM_ CD8 subtype.

We then further delineated the CD8 T cell population into memory subsets based on the expression of CD45RA and CCR7 surface markers, as described by the groups of Sallusto and Lanzavecchia (Figure 5A). Representative distributions of CD8 T cells in blood and endocervix of *C. trachomatis*-negative and *C. trachomatis*-positive women are shown in Figure 5B. Analyses of blood and endocervical CD8 T cell subtype percent- distribution from 16 women using repeated ANOVA indicated a significant tissue site interaction (p<0.0001; with Greenhouse-Geisser correction). Further, a paired comparison of mean percentage CD8 T cell subtypes in blood and endocervix, using t- test with confirmatory analysis using the Wilcoxon Signed Rank test, indicated a significantly higher percentage of T_EM_ cells in the endocervix (p<0.0001), with a significantly lower percentage of naïve cells (p<0.0001). Taken together these data indicate that, while the different CD8 T cell subtypes are relatively distributed in the blood, the endocervix is primarily populated by effector memory (T_EM_) CD8 T cells (Figure 5C and 5D).

### Effector memory CD8 T cells (T_EMs_) in the human endocervix have limited perforin expression

**Figure 6:**
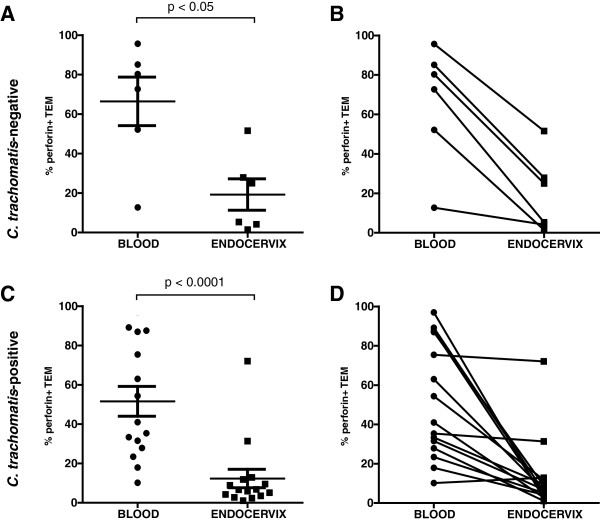
**Effector memory CD8 (TEM) cells in the endocervix are low in perforin.** (**A**) %perforin+ CD45RA^-^CCR7^-^CD8^+^ T cells T_EM_ in the blood and endocervix of *C. trachomatis*-negative (n=6) women, (**B**) a comparison of perforin expression in paired blood and endocervical derived T_EM_*C. trachomatis*-negative women, (**C**) %perforin+ of T_EM_ in the blood and endocervix of *C. trachomatis*-positive (n=15) women, (**D**) a comparison of perforin expression in paired blood and endocervical derived T_EM_*C. trachomatis*-positive women. Statistical analyses were performed using difference scores (blood – endocervix) and non-parametric Wilcoxon Rank Sum test.

Based on the observation that the endocervix is primarily populated by CD8 T_EM_ cells, we focused our subsequent analyses on this subpopulation. Here, the cytolytic potential of CD8 T_EM_ cells was assessed by analyzing flow cytometry data for the percentage of perforin expressing CD8 T_EM_ cells, using difference scores (blood – endocervix) and non-parametric analysis. Based on the Wilcoxon Rank Sum test, a significant difference in the percentage of perforin-expressing CD8 T_EM_ cells from blood and endocervix were observed in both *C. trachomatis*-negative (p<0.05) and in *C. trachomatis*-positive (p<0.0001) women as shown in Figures 6A and 6C respectively. Furthermore, pair-wise comparison of the percentage of perforin+ T_EM_ in the blood and endocervix of individual samples demonstrated that in both *C. trachomatis*-negative (Figure 6B) and *C. trachomatis*-positive patients (Figure 6D) there was a general trend for the endocervical T_EM_ to have lower percentage of perforin positive cells compared to T_EM_ derived from the blood. However, further analyses, with repeated measures ANOVA, using *C. trachomatis* infection status as a grouping factor, showed that although there were differences in blood vs. endocervix, no interaction with *C. trachomatis*-infection status was observed. These data show that despite the significant increase in CD8 T cells infiltrating the endocervix during *C. trachomatis*-infection, the relative low perforin level of the CD8 T T_EM_ cell population is still observed.

### IFN gamma exposure of epithelial cells could potentially mediate the downmodulation of perforin expression of T_EM_ CD8 T cells in the cervical microenvironment

**Figure 7:**
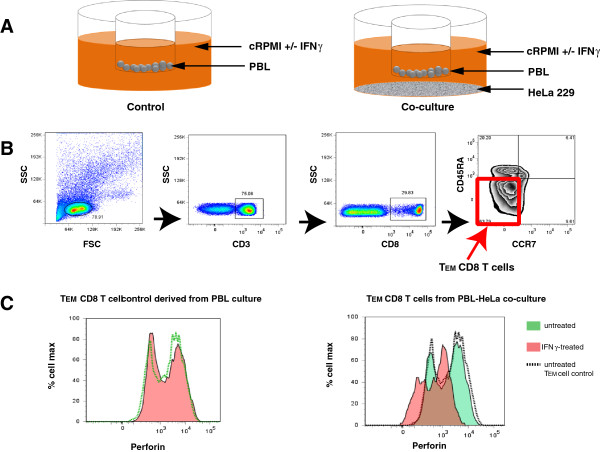
**IFN gamma could potentially induce a decrease in perforin of T**_**EM**_**CD8 T cells.** (**A**) HeLa 229 cervical epithelial cells and peripheral blood epithelial cells (PBL) co-culture set up. PBL were cultured alone in a transwell insert (left panel) or co-cultured with a monolayer of HeLa 229 cells (right panel) at the bottom of a well of a 12-well culture dish, in the presence or absence of 600 U/mL IFN gamma; (**B**) Gating strategy for CD8 T_EM_ cell population from PBL. The CD8 T_EM_ cells were identified by gating the cells based on FSC/SSC profile, followed by CD3, CD8 and CCR7 and CD45RA expressions; (**C**) Perforin expression of CD8 T_EM_ cell subpopulation from PBLs co-cultured with IFN gamma-exposed HeLa 229 cells. Histogram overlay of perforin expression of control CD8 T_EM_ cells derived from PBLs cultured alone in the presence (red) or absence (green) of IFN gamma is shown in the left panel and histogram overlay of CD8 TEM cells derived from PBL-HeLa 229 co-culture exposed (red) or not exposed (green) to IFN gamma is shown in the right. Dotted histogram overlay represents the untreated control T_EM_ perforin expression. The histograms are representative of results from two independent experiments.

Our *ex vivo* data suggested that endocervical CD8 T T_EM_ cells have a low perforin content. Therefore, we investigated one of the factors that could influence this phenotype. We hypothesized that IFN gamma could be one of the mediating factors that drives the decrease in perforin content of endocervical CD8 T_EM_ cells based on the following observations from previous studies: 1) IFN gamma levels in the female genital tract are elevated during the secretory stage of menstruation [16] and during decidualization in successful pregnancies [17]; 2) Animal models of *C. trachomatis* infections have demonstrated that T cells with the capacity to secrete IFN gamma migrate to the site of *Chlamydia* infection [18]; 3) High levels of IFN gamma are found in the FGT of *C. trachomatis*-infected patients [19]; and 4) IFN gamma can induce the expression of indoleamine-2,3-dioxygenase (IDO) in epithelial cells [20]; and IDO, a tryptophan catabolic enzyme, could downmodulate perforin expression in CD8 T cells [21]. Therefore, based on these previous findings, we developed a PBL-HeLa 229 cervical epithelial cell co-culture model to investigate whether the exposure of epithelial cells to IFN gamma has an effect on perforin expression by CD8 T_EM_ cells. To do this, PBLs were cultured alone or with a monolayer of HeLa 229 cells and with or without 600 U/mL of IFN gamma (Figure 7A). After 38 hours in culture, the PBLs were then stained to identify the different CD8 T cell subpopulations and intracellular perforin and granzyme expression. The gating strategy to delineate the different CD8 T cell subpopulation is shown in Figure 7B. This gating allowed us to identify the CD8 T_EM_ cell subpopulation from the PBLs, and to assess the perforin expression of these cells in the absence or presence of IFN gamma.

We found that the level of perforin expression by the control CD8 T_EM_ cells from PBLs cultured in the absence of HeLa 229 cells did not shift in the presence of IFN gamma (Figure 7C – left panel). However, when we co-cultured PBLs with HeLa cells, the addition of IFN gamma in the culture medium resulted in the negative shift in the fluorescence intensity of perforin, suggesting a downmodulation of the expression of this protein (Figure 7C – right panel). Therefore, our *in vitro* data suggest that IFN gamma could be potential mediator of the downmodulation of perforin expression in CD8 T_EM_ cells.

## Discussion

Using both *in situ* tissue-based and *ex vivo* cytobrush-sampling protocols we confirm here that CD8 T cells comprise a significant component of the endocervical T cell infiltrate during *C. trachomatis* infection. Moreover, we also report that endocervical CD8 T cells are primarily of the T_EM_ subtype, but, unlike their peripheral counterparts that express high levels of perforin, these cells have low levels of this protein. Since low perforin content is observed in CD8 T_EM_ cells from both normal and *C. trachomatis*-infected tissues we hypothesize that the endocervical milieu drives this phenotype. That the endocervical CD8 T_EM_ subpopulation is low in perforin may reflect an immunological response pattern that supports feto-maternal tolerance and facilitates a fine balance between protection of tissue integrity and defence against sexually transmitted pathogens.

Human studies and animal models of *C. trachomatis* infection have previously demonstrated recruitment of immune cells to the local site of infection. [18,22-29]. Compelling evidence for the ability of *Chlamydia*-specific CD8 T cells to migrate to infected murine FGT has been presented by Roan and Starnbach who generated retrogenic mice expressing a T cell receptor specific for CrpA, a *Chlamydia* specific T cell antigen [18]. They observed that adoptively-transferred *Chlamydia-*specific retrogenic CD8 T cells proliferated in genital tract-draining lymph nodes of naïve recipients in response to genital *C. trachomatis* challenge. Further, they also demonstrated that the adoptively transferred *Chlamydia*-specific CD8 T cells successfully migrated to the genital mucosa and acquired the ability to produce IFN gamma *[ibid]*. In our earlier study, in which we observed elevated endocervical T cell numbers during culture positive endocervical *C. trachomatis* infections, we also noted that T cells numbers significantly decline in these women post successful antibiotic treatment [30]. We interpret these observations to suggest that active *C. trachomatis* infection is most likely responsible for the recruitment of T cells at the site of infection. We also observed that CD8 T cells constitute a significant proportion of the T cell infiltrates found in the endocervix *[ibid]*. Thus, our data from human study, and reports based on animal studies mutually support the finding that CD8 T cells migrate into the FGT in response to *C. trachomatis* infection.

Intriguingly, although animal and human studies suggest that CD8 T cells are recruited to the site of *C. trachomatis* infection, the role of their cytolytic activity in protective immunity remains uncertain. Some studies suggest that CD8 T cells can play a role in clearance of *C. trachomatis* infection [26,31], while other studies implicate these cells to be responsible for immune-mediated pathology. As an example, Voorhis *et al.* found an association between perforin positive CD8 T cells and salpingeal tissue scarring in non-human primate model of *C. trachomatis* infection [32]. Further, in a study by Murthy *et al*., CD8 T cells were suggested to mediate oviduct pathology (hydrosalpinx) following *C. muridarum* infection in mice, but this was via their TNFα activity. This finding was demonstrated by the restoration of hydrosalpinx upon repletion of mice genetically deficient in CD8 T cells with perforin-deficient, but not with TNFα-deficient, CD8 T cells [33]. Therefore, currently, there is no direct evidence that perforin plays a role in immune-mediated pathology by CD8 T cells during *C. trachomatis* infection. Furthermore, evidence based on lung and genital infection studies in perforin-knockout mice suggests that perforin is not required for clearance of murine chlamydial infection [34]. Based on these animal models, we could surmise that the perforin-dependent CD8 T cell cytolytic response may not play a critical role in controlling *C. trachomatis* burden in the human endocervix.

While studies by others and us suggest a limited role for perforin-dependent CD8 T cell cytolytic activity against *C. trachomatis*, we do not discount the possibility that CD8 T cells may be involved in perforin-independent immune mechanisms. Particularly, we believe that during *C. trachomatis* infection in the human endocervix, CD8 T cells may significantly contribute to the accumulation of IFN gamma at this site as demonstrated in murine studies by Starnbach’s group [18,31]. This finding is supported by a report based on a human study that IFN gamma levels are elevated in the endocervix of *C. trachomatis*-infected women compared to uninfected controls [19].

Two of the possible mechanisms that could induce low perforin content in CD8 T_EM_ in *Chlamydia*-infected endocervix are discussed here. These are: 1) CD8 T_EM_ cells that have migrated to the endocervix in response to *C. trachomatis* infection exhibit active and continuous degranulation resulting in the loss of their perforin content, and; 2) The physiologic pressure in the female genital tract drives perforin downmodulation in CD8 T_EM_ cells. To our knowledge, the differences in degranulation sensitivity and the rates of granule protein recovery between CD8 T cells and NK cells are not yet well defined. However, we note here that unlike the CD8 T_EM_ cells, we observed that endocervical NK cells have a high perforin content (representative data shown in Figure 4), suggesting that our observations are not due to spontaneous and continuous degranulation of all cytolytic immune cells. Further, although not the focus of this study, we believe that our data suggests that NK cells could maintain a high cytolytic potential in the endocervix, and are likely to be a key immune cell population that mediates host cytolytic immune response to *C. trachomatis* infection. Consistent to this notion is our previous finding that the NK cell ligand expression in *C. trachomatis* infected endocervical epithelial cells renders these cells more susceptible to NK cell-mediated cytolysis [35].

To address the first possibility, a study on degranulation status of CD8 T cell infiltrates in the endocervix would need to be undertaken. One experimental approach to test this possibility would be the concurrent measurement of the surface expression of CD107a, a marker of degranulation [36], and the intracellular perforin content of CD8 T cells derived from the endocervix. In principle, the low perforin content of CD8 T cells in the endocervix of women could result from their degranulation in response to stimulation of resident *Chlamydia*-specific, and other pathogen – specific CD8 T cells at this site. As we have not tested for degranulation in this study, we do not discount that this is a plausible mechanism that could underlie the low perforin content of CD8 T cells in the endocervix. Therefore, further study needs to be undertaken to test this possibility.

The second possibility, that physiological pressure in the endocervix drives the low perforin content of CD8 T cells, was explored in this study. Specifically, we have provided a proof-of-principle that the IFN gamma exposure of cervical epithelial cells could potentially downmodulate perforin expression of CD8 T_EM_ cells in the same microenvironment. IFN gamma has been well established to induce indoleamine-2,3-dioxygenase (IDO) expression in epithelial cells. We also confirmed this in the HeLa 229 cells that were utilized in our *in vitro* study by immunoblotting HeLa 229 lysates with an IDO1-specific antibody (data not shown). IDO has been reported to impair the cytotoxic function of CD8 T cells by reducing perforin expression in these cells [21]. Therefore, IFN gamma secretion by infiltrating CD8 T cells during *C. trachomatis* infection may lead to an increase in the levels of IDO in the endocervix that could induce the decrease in perforin content of the local CD8 T_EM_ cells. While we have not yet been able to test this hypothesis *in vivo*, we believe that further investigation into IDO and perforin regulation in the endocervix during *C. trachomatis* infection is warranted to determine the mechanisms and functional significance of the low perforin content of CD8 T_EM_ cells in this unique anatomical region.

Interestingly, there are indications that our hypothesis, proposing that IFN gamma and IDO could mediate the downmodulation perforin expression of endocervical CD8 T cells during *C. trachomatis* infection, could be generally operant in the FGT even in the absence of chlamydial infection. The first series of studies that support our hypothesis includes the observation that endometrial tissues have high levels of IFN gamma at the secretory stage of the menstrual cycle but not during the proliferative phase [16]. Consistent with these findings are immunohistochemical and mRNA-based analyses demonstrating that IDO expression is low during the proliferative stage, but is elevated during the secretory phase [37,38]. Significantly, an elegant study by Wira’s group demonstrated that T cells isolated from tissues during proliferative phase of menstrual cycle demonstrate cytolytic capacity but those during the secretory phase do not [39]. Therefore, the presence of IFN gamma and elevated levels of IDO expression during secretory phase, which can downmodulate perforin expression in CD8 T cells, coincides with the dampening of cytolytic T cell activity.

The second series of studies that support our hypothesis is based on the analyses of IDO expression during pregnancy. Elevated IDO expression has been found in the epithelium of cervical glands, Fallopian tubes and endometrial stromal cells during decidualization in both animal and human studies [37,38,40,41]. Interestingly, CD8 T cells comprise the largest fraction of T cells at the fetal-maternal interface [42]. However, analysis of perforin expression of CD8 T cells during pregnancy revealed that while peripheral CD8 T cells express perforin, this cytolytic molecule is deficient in decidual CD8 T cells [43,44]. These finding is consistent with the studies by Mellor *et al.* suggesting that IDO-mediated T cell dysfunction plays a significant role in the induction of feto-maternal tolerance [45-48]. Therefore, it seems apparent that the IFN gamma-IDO-perforin axis is an important component of the regulation of CD8 T cell cytolytic activity in the female genital tract.

If our hypothesis that IFN gamma-mediated induction of IDO is the primary mechanism involved in lowering perforin content of CD8 T_EM_ cells in the endocervix, studies on the CD8 T cell immune repertoire of *C. trachomatis* infected women stratified based on their stage of menstrual cycle at the time of sample collection would be desirable, as this would clarify whether elevation of IFN gamma levels during *C. trachomatis* infection could overcome the low IDO expression during the proliferative stage. This would shed light on whether regardless of the stage of the menstrual cycle, the CD8 T_EM_ cells in the endocervix are consistently low in perforin content during *C. trachomatis* infection.

## Conclusions

The findings in this study suggest that perforin-dependent CD8 T cell-mediated cytoxicity may play a limited role in immune control of human endocervical *C. trachomatis* infection. However, further studies are needed to clearly establish the mechanism that drives the decrease in perforin levels in normal and *C. trachomatis*-infected endocervix. Nevertheless, despite the challenging nature of human studies in the female genital tract, we have generated *ex vivo* data that provides important insights into the endocervical immunological repertoire that may influence the outcome of *C. trachomatis* infection at this site.

## Methods

### Immunohistochemical assessment of CD8 T cell infiltrates

Archived endocervical tissue paraffin blocks were utilized to assess the perforin and granzyme expression of endocervical CD8 T cell infiltrates *in situ*. Collection of endocervical tissues was approved by Louisiana State University Health Sciences Center Institutional Review Board. The endocervical tissues were derived from biopsies or discarded hysterectomy specimen. All specimens were screened for the presence of inclusions by chlamydial LPS staining. A confirmatory test of chlamydial positivity was performed on 3 of the 4 chlamydial-LPS positive tissues by *ompA* genotyping as previously described [30]. Endocervical tissues positive for *C. trachomatis* infection (n=4) and endocervical tissues negative for *C. trachomatis* infection (n=6) were analyzed by immunohistochemical staining for CD3, CD8 and perforin expression. In each experiment, sections of cell pellet consisting of peripheral blood mononuclear cells buffered with HeLa 229 cells were used as a positive control, while staining with isotype-matched irrelevant antibodies were used as negative controls. Immunohistochemical staining was undertaken by first cutting 4 μM sections of tissue which were immediately fixed on glass slides. Paraffin wax was then removed by baking the slides at 65°C for 1 hour, after which the slides were soaked in Antigen Retrieval solution (Biocare) and heated in a de-cloaking chamber (Biocare) for 20 minutes. The processed sections of tissues on the slides were then blocked using Background Sniper blocking reagent (Biocare) to prevent non-specific background staining for 1 hour at room temperature. Individual tissue sections were then reacted with the following primary antibodies: Anti-CD3 (Dakocytomation), anti-CD8 (Biocare), anti-perforin (Biolegend) or isotype-matched control (BD Biosciences) solution and incubated for 1 hour. After several washes with phosphate buffered saline (PBS), the tissue sections were incubated with appropriate secondary-antibodies conjugated to alkaline phosphatase. The slides were then washed with PBS to remove unbound antibodies, and Vulcan Fast Red chromogen system (Biocare) was added and allowed to develop. After a substrate reaction was observed in positive controls, the slides were soaked in distilled water and counterstained with hematoxylin. Tissues were then dehydrated in a series of methanol and fixed on the slide by covering with coverslip with permanent mounting medium, VectaMount (Vector Laboratories). The differences in number of CD3 and CD8 T cell infiltrates were assessed using Student’s t-test after checking the assumptions of normality and homogeneity of variance, followed by confirmatory analyses with Wilcoxon Rank Sum test.

### Patient population

To assess of the cytolytic potential of CD8 T cells in the blood and endocervix, we recruited participants into a study through the Delgado Clinic, New Orleans, Louisiana. Written informed consent as approved by the Louisiana State University Health Sciences Center Institutional Review Board was obtained from each participant. A total of 21 paired blood and endocervical cell samples were collected from women 18–30 years old attending the clinic who were enrolled based on satisfying at least one of the following criteria: (1) clinical evidence of cervicitis, (2) having a male partner who was tested positive for *C. trachomatis* infection (CT+) and (3) a positive test result for *C. trachomatis* infection by nucleic acid amplification test (NAAT) 3 to 30 days prior to enrolment. The participants provided information on demographics, history of previous sexually transmitted infections, antibiotic usage, and sexual behaviour. The participants were also examined by a nurse practitioner for mucupurulent cervical and vaginal discharge, bleeding and presence of clue cells. Urine samples were obtained to test for *Neisseria gonorrhoea* and *C. trachomatis* infection by NAAT. The *C. trachomatis* positive population were defined by a positive C. trachomatis NAAT result and confirmed with a *C. trachomatis* positive culture. Additional vaginal swabs were taken to assess for *Trichomonas vaginalis*, yeast infection, and bacterial vaginosis as previously described [15]. HIV positive patients were excluded from the study.

### Sample collection and processing

To analyze the immune cell repertoire in the endocervix of women enrolled in the study, parallel samples of whole blood (40 mL) and endocervical cells collected using two cytobrushes were performed as previously described. Peripheral blood mononuclear cells (PBMC) were isolated by conventional ficoll differential centrifugation, and endocervical cells were isolated from the cytobrushes following the previously established protocol in our laboratory [30].

### Immunophenotyping by multiparameter flow cytometry

To analyze the cytolytic potential of systemic and endocervical CD8 T cells, polychromatic (6-color) flow cytometry was performed. After processing the PBMC and endocervical cells, aliquots of these cells were made. One aliquot of cells was stained with fluorescently labeled antibodies against surface markers: CD56-APC (BD Pharmingen), CD3-PerCP Cy5.5 (BD Biosciences), CD4-Alexa Fluor 700 (Biolegend), CD8-APC-Cy7 (BD Pharmingen). Second aliquot for Isotype controls was stained with isotype-matched fluorescent antibodies: APC IgG1k Isotype (BD Biosciences), PerCP Cy5.5 IgGk isotype (BD Biosciences), Alexa Flour 700 IgG1k isotype (Biolegend), APC-Cy7 IgG1k Isotype (BD Biosciences). The cells were then permeabilized using Cytofix/Cytoperm (BD Pharmingen), a formulation of paraformaldehyde and saponin, to fix and permeabilize the cells. Following permeabilization, the cells were stained for intracellular proteins using labeled antibodies against Perforin-FITC (Abcam) and Granzyme B-PE (Cell Sciences). The second isotype aliquot was stained with FITC IgG2b Isotype (BD Biosciences) and PE IgG1 Isotype (BD Biosciences). CD4 T cells and NK cells were also analyzed to serve as cellular controls. Granzyme was utilized as intracellular staining control. Using the polychromatic flow cytometry approach, the different immune cells were simultaneously identified and cytolytic potentials measured. The percentage of cells positive for perforin was determined by gating the cells based on the threshold fluorescence of the FITC IgG2b Isotype control.

### CD8 T cell phenotyping

To further analyze CD8 T cell subpopulations, a third aliquot of cell preparation were stained for CD3-PerCP Cy5.5 and CD8-APC Cy7 in conjunction with CCR7-APC (R&D Systems) and CD45 RA-Alexa Fluor 700 (AbD Serotec), perforin-FITC (Abcam) and granzyme B-PE (CellSciences). This is to categorize the different subpopulations of CD8 T cells following the classification by Sallusto *et al.*[8,9]. Further phenotyping of CD8 T cells allowed the identification of the different CD8 T cell subpopulation for a more extensive analysis of memory CD8 T cell population.

### PBL-HeLa 229 cervical epithelial cell co-culture

HeLa 229 cervical epithelial cells were obtained from the ATCC, and cultured in complete RPMI 1640 (cRPMI) culture medium with 10% human AB serum (Sigma); supplemented with 200 mM glutamine (Invitrogen) and 0.25% (wt/vol) glucose (Sigma). HeLa cells were seeded in a 12-well culture plate and cultivated for 24 to 36 hours until about 80% confluence was achieved. Peripheral blood mononuclear cells (PBMCs) were isolated from whole blood as described above. The isolated PBMCs were then placed in 6-well culture dish and incubated at 5% CO2 at 37°C for 4 hours to allow monocytes to adhere to the plastic. The non-adherent-PBLs were then collected, washed and counted using a heamocytometer. The PBLs were then placed on cell culture transwell insert on laid on top of a well on a 12 well plate without or with HeLa cells. The cells were cultured for 38 hours in the absence or presence of 600 U/mL IFN gamma in cRPMI. The cells were harvested, and the viability of the cells were assessed by trypan blue staining to confirm that ~95% of the cells are viable. The cells were then stained with monoclonal antibodies against CD3, CD8, CD45RA, CCR7, perforin and granzyme, and flow cytometric analyses were performed as described above.

### Statistical analyses

Data were summarized using means (+ standard deviations), medians, and percentages as appropriate. Normality assumptions were assessed using the Shapiro-Wilks test, and where they were violated, parametric analyses were confirmed with analogous nonparametric methods. Differences between levels of CD3 and CD8 cells in *C. trachomatis*-positive and *C. trachomatis*-negative tissues were tested using Student’s t-test (Wilcoxon Rank Sum test). Paired blood and endocervical specimens were assessed usi`ng paired t-test (Wilcoxon Signed Rank test), and repeated measures ANOVA (Signed Rank and Rank Sum tests) was used to determine if blood versus endocervical differences varied with *C. trachomatis* infection status (interaction). SAS statistical software (version 9.2) was used for all analyses.

## Competing interests

The authors declare that they have no competing interests.

## Authors’ contributions

JAI: performed all the assays, analyzed data, and wrote the manuscript. LM: participated in the design of the study and carried out the statistical analyses. CP: participated in flow cytometric data acquisition and analyses. ML: participated in immunohistochemical data acquisition and analyses. ST: participated in the design of the study and collection of samples. DHM: participated in the design and coordination of the study, helped to write the manuscript. AJQ: conceived the study, participated in the design and coordination and helped to write the manuscript. All authors read and approved the final manuscript.

## References

[B1] Smyth MJ Trapani JA Granzymes: exogenous proteinases that induce target cell apoptosis Immunol Today 1995 16 4 202 206 7734049 10.1016/0167-5699(95)80122-7

[B2] Trapani JA Jans DA Jans PJ Smyth MJ Browne KA Sutton VR Efficient nuclear targeting of granzyme B and the nuclear consequences of apoptosis induced by granzyme B and perforin are caspase-dependent, but cell death is caspase-independent J Biol Chem 1998 273 43 27934 27938 9774406 10.1074/jbc.273.43.27934

[B3] Uellner R Zvelebil MJ Hopkins J Jones J MacDougall LK Morgan BP Podack E Waterfield MD Griffiths GM Perforin is activated by a proteolytic cleavage during biosynthesis which reveals a phospholipid-binding C2 domain EMBO J 1997 16 24 7287 7296 9405358 10.1093/emboj/16.24.7287 PMC1170329

[B4] Tschopp J Schafer S Masson D Peitsch MC Heusser C Phosphorylcholine acts as a Ca2+−dependent receptor molecule for lymphocyte perforin Nature 1989 337 6204 272 274 2783478 10.1038/337272a0

[B5] Kagi D Ledermann B Burki K Seiler P Odermatt B Olsen KJ Podack ER Zinkernagel RM Hengartner H Cytotoxicity mediated by T cells and natural killer cells is greatly impaired in perforin-deficient mice Nature 1994 369 6475 31 37 8164737 10.1038/369031a0

[B6] Lanzavecchia A Sallusto F Understanding the generation and function of memory T cell subsets Curr Opin Immunol 2005 17 3 326 332 15886125 10.1016/j.coi.2005.04.010

[B7] Germain RN MHC-dependent antigen processing and peptide presentation: providing ligands for T lymphocyte activation Cell 1994 76 2 287 299 8293464 10.1016/0092-8674(94)90336-0

[B8] Sallusto F Lenig D Forster R Lipp M Lanzavecchia A Two subsets of memory T lymphocytes with distinct homing potentials and effector functions Nature 1999 401 6754 708 712 10537110 10.1038/44385

[B9] Sallusto F Geginat J Lanzavecchia A Central memory and effector memory T cell subsets: function, generation, and maintenance Annu Rev Immunol 2004 22 745 763 15032595 10.1146/annurev.immunol.22.012703.104702

[B10] Champagne P Dumont AR Sekaly RP Learning to remember: generation and maintenance of T-cell memory DNA Cell Biol 2001 20 12 745 760 11879568 10.1089/104454901753438561

[B11] Sutton VR Waterhouse NJ Baran K Browne K Voskoboinik I Trapani JA Measuring cell death mediated by cytotoxic lymphocytes or their granule effector molecules Methods 2008 44 3 241 249 18314055 10.1016/j.ymeth.2007.11.011

[B12] Masopust D Vezys V Wherry EJ Barber DL Ahmed R Cutting edge: gut microenvironment promotes differentiation of a unique memory CD8 T cell population J Immunol 2006 176 4 2079 2083 16455963 10.4049/jimmunol.176.4.2079

[B13] Shacklett BL Cox CA Quigley MF Kreis C Stollman NH Jacobson MA Andersson J Sandberg JK Nixon DF Abundant expression of granzyme A, but not perforin, in granules of CD8+ T cells in GALT: implications for immune control of HIV-1 infection J Immunol 2004 173 1 641 648 15210827 10.4049/jimmunol.173.1.641

[B14] Rukavina D Laskarin G Rubesa G Strbo N Bedenicki I Manestar D Glavas M Christmas SE Podack ER Age-related decline of perforin expression in human cytotoxic T lymphocytes and natural killer cells Blood 1998 92 7 2410 2420 9746781

[B15] Arnold V Balkow S Staats R Matthys H Luttmann W Virchow JC Jr Increase in perforin-positive peripheral blood lymphocytes in extrinsic and intrinsic asthma Am J Respir Crit Care Med 2000 161 1 182 186 10619818 10.1164/ajrccm.161.1.9902104

[B16] Kumar S Li Q Dua A Ying YK Bagchi MK Bagchi IC Messenger ribonucleic acid encoding interferon-inducible guanylate binding protein 1 is induced in human endometrium within the putative window of implantation J Clin Endocrinol Metab 2001 86 6 2420 2427 11397834 10.1210/jcem.86.6.7534

[B17] Vives A Balasch J Yague J Quinto L Ordi J Vanrell JA Type-1 and type-2 cytokines in human decidual tissue and trophoblasts from normal and abnormal pregnancies detected by reverse transcriptase polymerase chain reaction (RT-PCR) Am J Reprod Immunol 1999 42 6 361 368 10622466 10.1111/j.1600-0897.1999.tb00113.x

[B18] Roan NR Starnbach MN Antigen-specific CD8+ T cells respond to Chlamydia trachomatis in the genital mucosa J Immunol 2006 177 7974 7979 17114470 10.4049/jimmunol.177.11.7974

[B19] Arno JN Ricker VA Batteiger BE Katz BP Caine VA Jones RB Interferon-gamma in endocervical secretions of women infected with Chlamydia trachomatis J Infect Dis 1990 162 1385 1389 2121840 10.1093/infdis/162.6.1385

[B20] Feng GS Taylor MW Interferon gamma-resistant mutants are defective in the induction of indoleamine 2,3-dioxygenase Proc Natl Acad Sci USA 1989 86 7144 7148 2506547 10.1073/pnas.86.18.7144 PMC298012

[B21] Liu H Liu L Bizargity P Hancock WW Visner GA Reduced cytotoxic function of effector CD8+ T cells is responsible for indoleamine 2,3-dioxygenase-dependent immune suppression J Immunol 2009 183 1022 1031 19564344 10.4049/jimmunol.0900408

[B22] Olive AJ Gondek DC Starnbach MN CXCR3 and CCR5 are both required for T cell-mediated protection against C. trachomatis infection in the murine genital mucosa Mucosal Immunol 4 2 208 216 20844481 10.1038/mi.2010.58 PMC3010299

[B23] Rank RG Bowlin AK Kelly KA Characterization of lymphocyte response in the female genital tract during ascending Chlamydial genital infection in the guinea pig model Infect Immun 2000 68 9 5293 5298 10948157 10.1128/iai.68.9.5293-5298.2000 PMC101791

[B24] Morrison SG Morrison RP In situ analysis of the evolution of the primary immune response in murine Chlamydia trachomatis genital tract infection Infect Immun 2000 68 5 2870 2879 10768984 10.1128/iai.68.5.2870-2879.2000 PMC97499

[B25] Starnbach MN Bevan MJ Lampe MF Murine cytotoxic T lymphocytes induced following Chlamydia trachomatis intraperitoneal or genital tract infection respond to cells infected with multiple serovars Infect Immun 1995 63 9 3527 3530 7642287 10.1128/iai.63.9.3527-3530.1995 PMC173488

[B26] Starnbach MN Bevan MJ Lampe MF Protective cytotoxic T lymphocytes are induced during murine infection with Chlamydia trachomatis J Immunol 1994 153 11 5183 5189 7525725

[B27] Kelly KA Wiley D Wiesmeier E Briskin M Butch A Darville T The combination of the gastrointestinal integrin (alpha4beta7) and selectin ligand enhances T-Cell migration to the reproductive tract during infection with Chlamydia trachomatis Am J Reprod Immunol 2009 61 6 446 452 19392980 10.1111/j.1600-0897.2009.00705.x PMC2891913

[B28] Kelly KA Rank RG Identification of homing receptors that mediate the recruitment of CD4 T cells to the genital tract following intravaginal infection with Chlamydia trachomatis Infect Immun 1997 65 12 5198 5208 9393816 10.1128/iai.65.12.5198-5208.1997 PMC175749

[B29] Hawkins RA Rank RG Kelly KA Expression of mucosal homing receptor alpha4beta7 is associated with enhanced migration to the Chlamydia-infected murine genital mucosa in vivo Infect Immun 2000 68 10 5587 5594 10992458 10.1128/iai.68.10.5587-5594.2000 PMC101510

[B30] Ficarra M Ibana JS Poretta C Ma L Myers L Taylor SN Greene S Smith B Hagensee M Martin DH A distinct cellular profile is seen in the human endocervix during Chlamydia trachomatis infection Am J Reprod Immunol 2008 60 5 415 425 18798835 10.1111/j.1600-0897.2008.00639.x PMC2574558

[B31] Lampe MF Wilson CB Bevan MJ Starnbach MN Gamma interferon production by cytotoxic T lymphocytes is required for resolution of Chlamydia trachomatis infection Infect Immun 1998 66 11 5457 5461 9784557 10.1128/iai.66.11.5457-5461.1998 PMC108683

[B32] Van Voorhis WC Barrett LK Sweeney YT Kuo CC Patton DL Repeated Chlamydia trachomatis infection of Macaca nemestrina fallopian tubes produces a Th-1-like cytokine response associated with fibrosis and scarring Infect Immun 1997 65 2175 2182 9169748 10.1128/iai.65.6.2175-2182.1997 PMC175300

[B33] Murthy AK Li W Chaganty BK Kamalakaran S Guentzel MN Seshu J Forsthuber TG Zhong G Arulanandam BP Tumor necrosis factor alpha production from CD8+ T cells mediates oviduct pathological sequelae following primary genital Chlamydia muridarum infection Infect Immun 79 7 2928 2935 21536799 10.1128/IAI.05022-11 PMC3191981

[B34] Perry LL Feilzer K Hughes S Caldwell HD Clearance of Chlamydia trachomatis from the murine genital mucosa does not require perforin-mediated cytolysis or Fas-mediated apoptosis Infect Immun 1999 67 3 1379 1385 10024585 10.1128/iai.67.3.1379-1385.1999 PMC96471

[B35] Ibana JA Aiyar A Quayle AJ Schust DJ Modulation of MICA on the surface of Chlamydia trachomatis-infected endocervical epithelial cells promotes NK cell-mediated killing FEMS Immunol Med Microbiol 65 1 32 42 22251247 10.1111/j.1574-695X.2012.00930.x PMC5029121

[B36] Betts MR Brenchley JM Price DA De Rosa SC Douek DC Roederer M Koup RA Sensitive and viable identification of antigen-specific CD8+ T cells by a flow cytometric assay for degranulation J Immunol Methods 2003 281 1–2 65 78 14580882 10.1016/s0022-1759(03)00265-5

[B37] Sedlmayr P Blaschitz A Wintersteiger R Semlitsch M Hammer A MacKenzie CR Walcher W Reich O Takikawa O Dohr G Localization of indoleamine 2,3-dioxygenase in human female reproductive organs and the placenta Mol Hum Reprod 2002 8 4 385 391 11912287 10.1093/molehr/8.4.385

[B38] Kudo Y Hara T Katsuki T Toyofuku A Katsura Y Takikawa O Fujii T Ohama K Mechanisms regulating the expression of indoleamine 2,3-dioxygenase during decidualization of human endometrium Hum Reprod 2004 19 5 1222 1230 15070879 10.1093/humrep/deh218

[B39] White HD Crassi KM Givan AL Stern JE Gonzalez JL Memoli VA Green WR Wira CR CD3+ CD8+ CTL activity within the human female reproductive tract: influence of stage of the menstrual cycle and menopause J Immunol 1997 158 6 3017 3027 9058841

[B40] Drenzek JG Breburda EE Burleigh DW Bondarenko GI Grendell RL Golos TG Expression of indoleamine 2,3-dioxygenase in the rhesus monkey and common marmoset J Reprod Immunol 2008 78 2 125 133 18490060 10.1016/j.jri.2008.03.005

[B41] von Rango U Krusche CA Beier HM Classen-Linke I Indoleamine-dioxygenase is expressed in human decidua at the time maternal tolerance is established J Reprod Immunol 2007 74 1–2 34 45 17321596 10.1016/j.jri.2006.11.001

[B42] Tilburgs T van der Mast BJ Nagtzaam NM Roelen DL Scherjon SA Claas FH Expression of NK cell receptors on decidual T cells in human pregnancy J Reprod Immunol 2009 80 1–2 22 32 19394706 10.1016/j.jri.2009.02.004

[B43] Tilburgs T Scherjon SA Roelen DL Claas FH Decidual CD8+CD28- T cells express CD103 but not perforin Hum Immunol 2009 70 2 96 100 19150377 10.1016/j.humimm.2008.12.006

[B44] Tilburgs T Schonkeren D Eikmans M Nagtzaam NM Datema G Swings GM Prins F van Lith JM van der Mast BJ Roelen DL Human decidual tissue contains differentiated CD8+ effector-memory T cells with unique properties J Immunol 185 7 4470 4477 20817873 10.4049/jimmunol.0903597

[B45] Munn DH Zhou M Attwood JT Bondarev I Conway SJ Marshall B Brown C Mellor AL Prevention of allogeneic fetal rejection by tryptophan catabolism Science 1998 281 5380 1191 1193 9712583 10.1126/science.281.5380.1191

[B46] Mellor AL Sivakumar J Chandler P Smith K Molina H Mao D Munn DH Prevention of T cell-driven complement activation and inflammation by tryptophan catabolism during pregnancy Nat Immunol 2001 2 1 64 68 11135580 10.1038/83183

[B47] Mellor AL Munn DH Extinguishing maternal immune responses during pregnancy: implications for immunosuppression Semin Immunol 2001 13 4 213 218 11437628 10.1006/smim.2000.0317

[B48] Mellor AL Munn DH Tryptophan catabolism prevents maternal T cells from activating lethal anti-fetal immune responses J Reprod Immunol 2001 52 1–2 5 13 11600174 10.1016/s0165-0378(01)00118-8

